# The complete chloroplast genome of *Cinnamomum camphora* (L.) Presl., a unique economic plant to China

**DOI:** 10.1080/23802359.2019.1640083

**Published:** 2019-07-13

**Authors:** Peng Li, Guolun Jia, Guiliang Xin, Xia Cai

**Affiliations:** School of Life Science, Northwest University, Xi’an, Shaanxi, China

**Keywords:** Chloroplast genome, Illumina sequencing, phylogenetic analysis, *Cinnamomum camphora*, Lauraceae

## Abstract

*Cinnamomum camphora* (Lauraceae) Presl. is a unique economic plant to China. The complete chloroplast (cp) genome was sequenced and assembled by using Illumina paired-end reads data. The circular cp genome is 152,729 bp in size, including a pair of inverted repeat (IRs) regions of 20,074 bp, a large single copy (LSC) region of 93,688 bp and a small single copy (SSC) region of 18,893 bp. The genome contains 127 unique genes, including 83 protein-coding genes (PCGs), 36 transfer RNA genes (tRNAs), and 8 ribosomal RNA genes (rRNAs). Besides, 19 genes possess a single intron, while another three genes (ycf3, rps12, and clpP) have a couple of introns. The GC content of entire *C. camphora* cp genome, LSC, SSC, and IR regions are 39.2, 38.0, 33.9, and 44.4%, respectively. Phylogenetic analysis based on the concatenated coding sequences of cp PCGs showed that *C. camphora* and *Cinnamomum verum* are closely related with each other within the genus of *cinnamomum*.

*Cinnamomum camphora* (L.) Presl. belongs to the family Lauraceae and is a unique economic plant (Brewbaker 1959) and valuable traditional medicinal plant to china. *Cinnamomum camphora* contains many chemicals, among which borneol and camphor are well known bicyclic monoterpenoids and have been widely used in food, senior aromatic spices (Yang et al. 2018). For a long time, *C. camphora* has been used to treat inflammation related diseases, including sprains, rheumatic arthritis, abdominal pain, cough, and bronchitis (Li et al. 2018). Genome information of *C. camphora* has been poorly studied.

Fresh leaves of *C. camphora* were collected from the National Forest Park of Fuzhou (26°5′N, 119°17′E; Fujian, China). A voucher specimen (AC190121) is deposited at the Pharmacognosy Laboratory in Northwest University. Total genomic DNA was extracted with the modified CTAB method (Doyle and Doyle 1987) and constructed a shotgun library by next-generation sequencing on the Illumina Hiseq X Ten Sequencing System (Illumina, San Diego, CA, USA). 84,923 clean reads were used for the assembly of cp genome using the program MITObim v1.8 (https://github.com/chrishah/MITObim) (Hahn et al. 2013), with that of *Cinnamomum micranthum* (GenBank: KT833081) as the initial reference. The map of the complete cp genome was generated using the web-based tool OGDRaw v1.2 (http://ogdraw.mpimp-golm.mpg.de/) (Lohse et al. 2013). The complete cp genome sequence has been submitted to GenBank under the accession number MH356726.

The complete cp genome of *C. camphora* is a circular and double-stranded DNA molecule of 152,729 bp in length with a typical quadripartite structure, containing two inverted repeat (IRs) regions of 20,074 bp separated by a large single copy (LSC) region of 93,688 bp and a small single copy (SSC) region of 18,893 bp (Figure 1). It encodes 127 complete genes, including 83 protein-coding genes (79 PCG species), 36 transfer RNA genes (30 tRNA species) and 8 ribosomal RNA genes (4 rRNA species). Many genes occur in a single copy, only 15 genes species (*rps7*, *rps12*, *ycf1*, *ycf2*, *ndhB*, *trnA-UGC*, *trnI-GAU*, *trnL-CAA*, *trnN-GUU*, *trnR-ACG*, *trnV-GAC*, *rrn4.5*, *rrn5*, *rrn16* and *rrn23*) occur in double copies. 8 PCG genes (*atpF, ndhA, ndhB*, *rps16*, *rpl2*, *rpoC1*, *petB,* and *ycf68*) and 6 tRNA genes (*trnA-UGC*, *trnG-UCC*, *trnI-GAU*, *trnK-UUU*, *trnL-UAA,* and *trnV-UAC*) harbor a single intron, while three other genes (*clpP*, *rps12,* and *ycf3*) possess two introns. The overall GC content of *C. camphora* cp genome is 39.2%, while the corresponding values of the LSC, SSC, and IR regions are 38.0, 33.9 and 44.4%, respectively.

**Figure 1.**
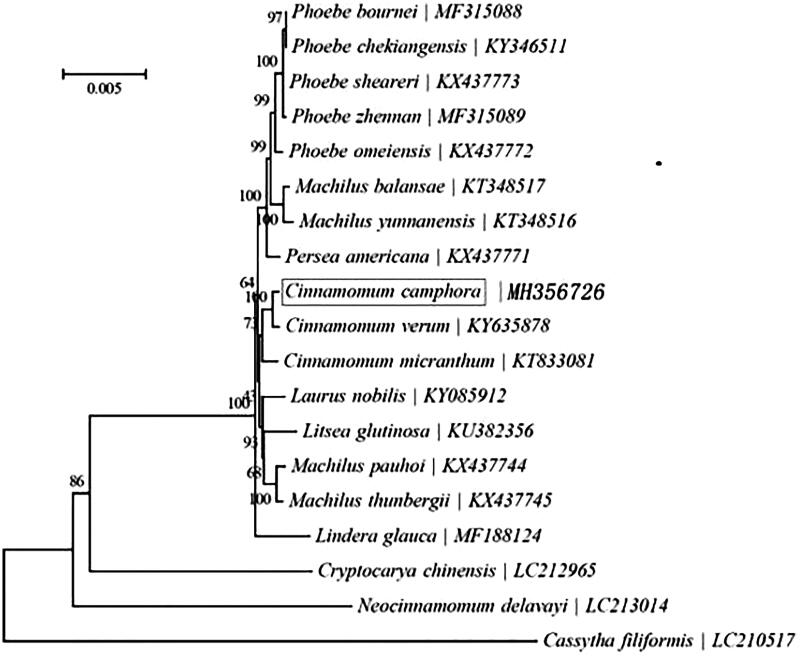
Phylogeny of 13 species within the order Lauraceae based on the neighbor-joining (NJ) analysis of chloroplast PCGs. The bootstrap values were based on 1000 resamplings and are placed next to the branches.

To investigate the phylogenetic position of *C. camphora*, a neighbour-joining (NJ) phylogenetic tree (Figure 1) was made based on the concatenated coding sequences of cp PCGs for 18 plastid genomes from published species of Lauraceae using MEGA7 with 1000 bootstrap replicates (Kumar et al. 2016) (http://www.megasoftware.net/). The result of the phylogenetic analysis shows that *C. camphora* is closely related to the species of *Cinnamomum verum*. The complete cp genome sequence adds valuable information for the study of the genetic diversity of *C. camphora* and Lauraceae.
